# X-ray microanalysis of dentine in primary teeth diagnosed Dentinogenesis Imperfecta type II

**DOI:** 10.1007/s40368-018-0392-2

**Published:** 2019-12-10

**Authors:** N. Sabel, J. G. Norén, A. Robertson, D. H. Cornell

**Affiliations:** 1 Department of Pediatric dentistry, Institute of Odontology, Sahlgrenska Academy, University of Gothenburg, Gothenburg, Sweden; 2 Department of Earth Sciences, University of Gothenburg, P.O. Box 460, SE 405 30 Gothenburg, Sweden

**Keywords:** Dentinogenesis imperfecta, Elemental composition, Primary tooth, X-ray microanalysis

## Abstract

**Aim:**

To analyse the elemental composition of dentine in primary teeth from children diagnosed with Dentinogenesis Imperfecta type II (DI) and from normal sound primary teeth using X-ray microanalysis.

**Materials and methods:**

X-ray microanalysis of the elements C, O, Na, Mg, P, Cl, K and Ca were performed in the dentine of five normal primary teeth and in seven primary teeth diagnosed DI. The analysis was made in a low magnification in 10 points from the enamel-dentine junction/root surface toward the pulp. The data was also evaluated with an inductive analysis.

**Results:**

Lower values for C were found in DI-dentine compared with normal dentine. Na had significantly higher values in DI-dentine while Mg had significantly lower values. The inductive analysis revealed that Na and Mg were the most important elements for discriminating DI-dentine from normal dentine.

**Conclusions:**

Dentine in primary teeth from patients diagnosed with Dentinogenesis Imperfecta type II analysed with XRMA have lower values of C and Mg and higher values of O and Na compared with normal primary dentine.

**Electronic supplementary material:**

The online version of this article (10.1007/s40368-018-0392-2) contains supplementary material, which is available to authorized users.

## Introduction

Dentinogenesis Imperfecta (DI) is an inherited single-gene disorder affecting the dentine which is classified into two main groups, DI type I and DI type II (Shields et al. 1973; Seow 2014). Differences in the pathological features are described, however, they are proposed to reflect variations in severity (Pallos et al. 2001; Kim et al. 2004; Kim and Simmer 2007; Barron et al. 2008). In several papers the genetics, clinical appearance and molecular aspects have been reported (Beattie et al. 2006; Hart and Hart 2007; Kim and Simmer 2007; Barron et al. 2008; Bailleul-Forestier et al. 2008a, b).

Clinically teeth affected by DI type II have an opalescent appearance with a colour varying from greyish to brown with blue streaks and often enamel is chipped off at the enamel-dentine junction (Shields et al. 1973; Levin et al. 1983; McKnight et al. 2008; Leal et al. 2010). Since the enamel is normal regarding its structure and mineralisation the colour of the teeth is a result of the colour of the underlying dentine. In DI type II, X-ray radiographs reveal pulp chambers which progressively obliterate, resulting in a denser dentine structure enhancing the deviation of colour of the teeth. The obliterated dentine is irregular and atypical compared with normal dentine (Waltimo 1994; Leal et al. 2010). The defect dentine in DI type II has been attributed to the weakness in the attachment between enamel and dentine creating the problems of chipping off of the enamel at the enamel-dentine junction (Gallusi et al. 2006; Wieczorek and Loster 2013).

In histo-morphological studies of the dentine in primary teeth with DI type II vascular inclusions have been described (Waltimo 1994; Lindau et al. 1999). X-ray microtomography has shown tubular structures in the dentine coinciding with the path of normal dentinal tubules but not continuous tubules (Davis et al. 2015). The dysfunctional mineralisation of the dentine and obliteration of the pulp evidently leaves blood vessels in the dentin which have been tied off and in the un-decalcified sections appear as vacuoles. The mineral concentration in DI type II dentine has been shown to be significantly lower compared to what was found in normal dentine and with needle-like crystallites indicating lack of intrafibrillar mineral (Kinney et al. 2001).

Reports of elemental analyses of the dentine in Dentinogenesis Imperfecta are few (Kerebel et al. 1981; Wieczorek and Loster 2012) and therefore the aim of this study was to analyse the elemental composition of dentine in primary teeth from children with DI and from normal sound primary teeth using X-ray microanalysis (XRMA).

## Materials and methods

### Tooth material

The tooth material comprised of seven exfoliated and extracted primary teeth (one mandibular incisor; one maxillary canine, five mandibular molar roots) from four patients diagnosed with Dentinogenesis Imperfecta type II. As reference teeth served five exfoliated primary incisors from five individuals with no known medical history. The teeth were collected through the years at the Department of Paediatric Dentistry, Institute of Odontology at the Sahlgrenska Academy, Göteborg, Sweden. The exfoliated and extracted teeth were collected when the children were between seven and 10 years of age, however, the exact dates are not known. The teeth were originally stored under cold conditions in 10% neutral buffered formaldehyde and transferred to 70% ethanol before embedding.

Prior to the XRMA analysis or embedding macro photos of the teeth were taken in a Leica M80 stereo microscope (Leica Mikrosysteme Vertrieb GmbH, Wetzlar, Germany) using a Leica digital camera (Leica DFC420 C, Leica Mikrosysteme Vertrieb GmbH, Wetzlar, Germany) with Leica Application Suite LAS V3.7.0 (Leica Microsystems AG, Heerbrugg, Switzerland).

### Ethical considerations

During the time period (around 1990–1996) when the teeth were collected, no approval for collecting teeth was necessary from the local Ethical Committee. The patient and the parents were informed regarding the use of the teeth for histological analyses and all teeth were donated of their own free will giving consent for any further examinations. The collected teeth were stored in a small glass flask which was marked with diagnoses and consecutively with a number in order to assure that the stored teeth derived from the same patient. There were no possibilities to identify any particular tooth with any patient.

### Embedding and cutting of teeth

The teeth were oriented for sagittal cutting in bucco-lingual direction and embedded in an epoxy-resin (Epofix®, Electron Microscopy Sciences, Fort Washington, PA, USA) using embedding moulds with a diameter of 15 mm. Each tooth was cut into two halves in a Leica SP1600 Saw Microtome (Leica Mikrosysteme Vertrieb GmbH, Wetzlar, Germany). The cut surface of the specimens was polished with polishing paper (grit 1200, 2400 and 4000) under water cooling. Prior to any analyses the surface was cleaned in an ultra-sonic bath in de-ionised water for 30 s.

### Light microscopy

**Fig. 1:**
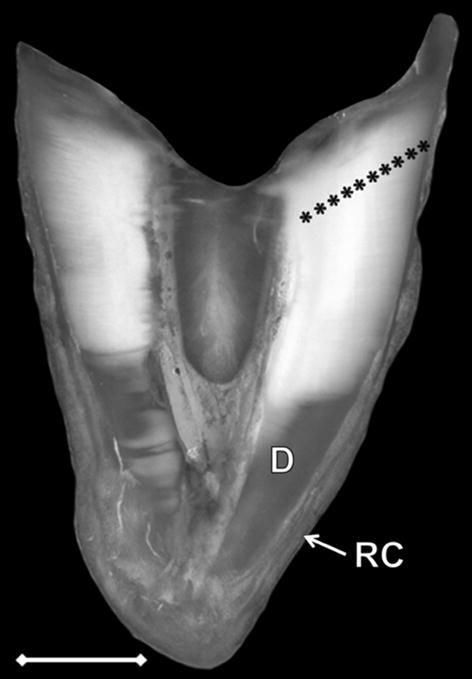
Un-decalcified specimen of a primary tooth from a patient diagnosed with Dentinogenesis Imperfecta type II with polished surface prepared for XRMA analysis. The principle for the locations of XRMA measurements are shown as black stars in which analysis in the 10 points was performed. (*D* dentine, *RC* root cementum, White bar = 2 mm)

Overviews of cut and polished specimens were taken in a Leica M80 stereo microscope (Leica Mikrosysteme Vertrieb GmbH, Wetzlar, Germany) at low magnification (0.75x) in incident light using a Leica digital camera (Leica DFC420) with Leica Application Suite LAS V3.7.0. (Leica Mikrosysteme Vertrieb GmbH, Wetzlar, Germany). The overviews were used for orientation for the X-ray microanalysis (Fig. 1).

### X-ray microanalyses

The polished samples were coated with gold in a plasma coater with a thickness of ≈25 nm. The XRMA analysis was performed in a Hitachi VP-SEM S-3400N (Hitachi, Tokyo, Japan) equipped with an Oxford EDS system and INCA Energy software (Oxford Instruments, Abingdon, UK). All analyses were carried out at 20 kV accelerating voltage and the working distance from sample to electron optical column was 9.6 mm, with a tolerance of ± 0.1 mm. The beam was aligned in “Microscope setup” using “Wave” in INCA when the microscope was positioned at a Faraday cage in the specimen holder. The beam current was adjusted to 6.0 nA ± 0.1nA, and was then checked every second hour.

The session calibration was performed using cobalt metal in the specimen holder in “Energy” setting of INCA and was made after acquiring a spectrum with a live time of 40 s. The Oxford EDS spectrometer was calibrated for each element at regular intervals using pure metal and simple oxide standards linked to the energy and peak area calibrations of cobalt and checked using Smithsonian Institute mineral standards.

For the elemental analysis of C, O, Na, Mg, P, Cl, K and Ca, the “All elements” and “Normalized” option in the INCA software was used and a spectrum was acquired with a live time of 100 s. The measurements were carried out in a low magnification (x45). After defining a line parallel to the dentinal tubules, the acquisition of data was automatically performed in 10 points from the enamel-dentine junction/root surface toward the pulp (Fig. 1). In the teeth which were completely obliterated the 10th point was located in the middle of the section.

The number of line measurements and total number of measurements in the dentine of the five normal sound primary teeth and in the seven primary teeth diagnosed with Dentinogenesis Imperfecta type II are shown in Table 1.

**Table 1:** Number of line measurements (**No. L**.) and total number of measurements (**Tot. no. M**.) in the dentine of five normal sound primary teeth (**N-01**—**N-05**) and in seven primary teeth from patients diagnosed with Dentinogenesis Imperfecta type II (**DI-01**—**DI-07**)

Sample	No. L.	Tot. no. M.
Normal		
N-01	4	40
N-02	2	20
N-03	4	40
N-04	2	20
N-05	4	40
DI		
DI-01	4	40
DI-02	6	60
DI-03	4	40
DI-04	5	50
DI-05	2	20
DI-06	3	30
DI-07	2	20

### Statistical analyses

The statistical analyses were performed using the IBM SPSS Statistics for Windows, Version 21.0. (IBM Corp., Armonk, NY, USA) employing the non-parametric Mann–Whitney U Test for independent samples for each point of location for the XRMA measurements. The level of significance was set to p < 0.05.

### Inductive analysis

An inductive analysis was performed to elucidate any relationship between the elements measured with XRMA and their origin, normal dentine or DI-dentine. All data was compiled in an Excel spread sheet, where the values (*numerical*) for the different elements (*attributes*) were set in columns, each row representing one point of measurement—one example. As *outcome*, a column of values (*discrete*) representing measurements from DI or normal dentine was added in order to enable recognition of any relationship between the different elements and the outcome values. The data was imported to the inductive analysis program XpertRule Analyser (Attar Software, Lancashire, UK). Before the analysis was performed, the discrete attribute was set as outcome. The results are presented in a hierarchic diagram (*knowledge tree*) in which the importance of every attribute in the inductive analysis is specified by its position in the knowledge tree. The higher in the tree, the more important for the outcome, and thus, the tree shows how different attributes affect the outcome.

An inductive analysis was performed using values for C, O, Na, Mg, P, Cl, K and Ca from the XRMA analysis with “*normal dentine*” and “*DI-dentine*” as discrete outcome values. In the analysis, 50% of the examples were randomly selected by the program for use in the induction of a knowledge tree (*training set*), the remaining examples were used for verification of the generated rules (*test set*). In the Verify option of the Analyser, a table will show the accuracy of the rules induced in the knowledge tree.

The option “*pruning*” in XpertRule Analyser presents an opportunity to reduce, for example, the effects of noise in the induced knowledge tree, an option which is particularly useful when an induced tree becomes too extensive. For discrete outcome values, a statistical pruning based on Chi square test of independence and the branches in the tree will be pruned (*cut*). The pruned knowledge tree can then be verified and the results can be compared with the non-pruned tree. An advantage is that a pruned knowledge tree will be much more feasible to interpret.

## Results

### Macro photos

**Fig. 2:**
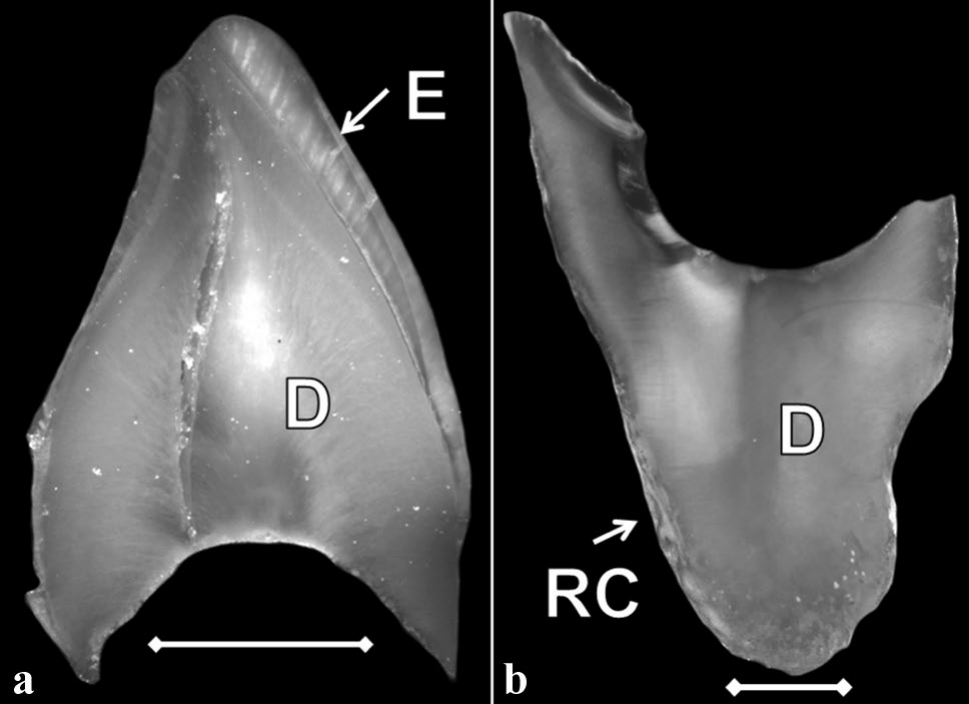
**a–b** Un-decalcified specimens of primary teeth diagnosed Dentinogenesis Imperfecta type II seen in incident light. **a** Primary incisor with obliterated pulp chamber. **b** Root of primary mandibular molar. (*E* enamel, *D* = dentine, *RC* root cementum; White bar = 2 mm)

The normal sound primary teeth appeared with a whitish colour while the crowns of the DI-teeth had a slight brownish colour. Some of the DI-teeth were broken by dryness sagittally in halves as and the exposed dentine appeared compact without any normal dentinal structure showing no alignment of dentinal tubules (Fig. 2a–b). The colour of the exposed dentine was yellowish to brownish shade appearing to be darker compared with the dentine in the normal primary teeth. Normal physiological resorption of the roots before exfoliation was seen in the normal primary teeth in contrast to what was seen the DI-teeth. In the extracted DI-teeth the coronal part was abraded and the pulp chambers in the roots were completely obliterated (Fig. 2a–b).

### XRMA results

The full set of data from the XRMA measurements in the dentine of normal primary teeth and in DI-dentine are given in Appendix 1.

The mean values and standard deviations for intensity corrections from the XRMA analyses of dentine in normal primary teeth and in DI-dentine for the elements C, O, Na, Mg, P, Cl, K and Ca are given in Table 2.

**Table 2:** Mean values (**Mean**) and standard deviations (**SD**) for intensity corrections from XRMA analyses of dentine in normal sound primary teeth (**N**) and in dentine in primary teeth from patients diagnosed with Dentinogenesis Imperfecta type II (**DI**) for the elements **C, O, Na, Mg, P, Cl, K** and **Ca**

	N		DI	
	Mean	SD	Mean	SD
**C**	0.21	0.014	0.21	0.029
**O**	0.17	0.011	0.18	0.020
**Na**	0.67	0.007	0.66	0.015
**Mg**	0.66	0.006	0.66	0.013
**P**	0.96	0.007	0.96	0.014
**Cl**	1.00	0.004	1.01	0.008
**K**	1.02	0.007	1.02	0.015
**Ca**	0.92	0.0023	0.92	0.006

Intensity corrections compensate for the matrix effects of the analysed sample and are calculated from the element composition. The apparent concentrations are divided by the intensity corrections to produce the true concentrations

### The elements C, O, Na, Mg, P, Cl, K, Ca and the ratio Ca/P (Fig. 3a–i)

The concentration profiles for the elements C, O, P, Cl, K and Ca were parallel to each other with only minor differences between normal and DI-dentine.

**Fig. 3:**
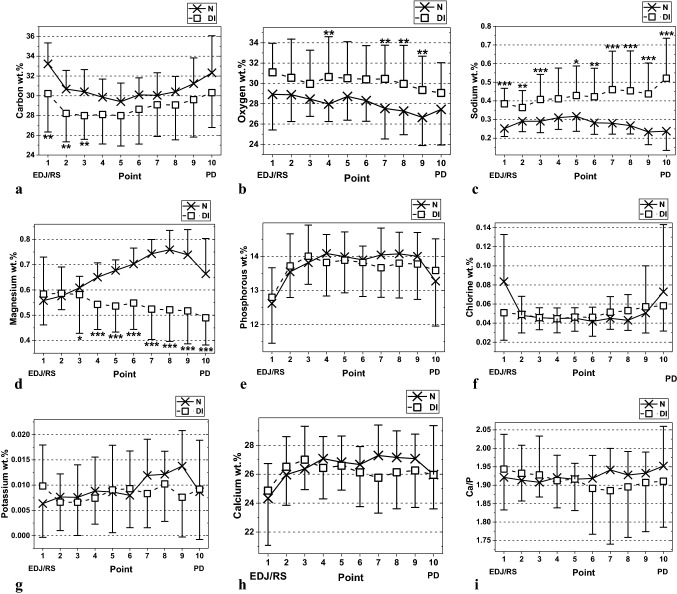
**a–i** Semi-quantitative mean values and standard deviations for **C, O, Na, Mg, P, Cl, K, Ca** and the ratio **Ca/P** from XRMA measurements in dentine from normal primary teeth (**N**) and from primary teeth diagnosed with Dentinogenesis Imperfecta type II (**DI**). The measurements are carried out from the enamel-dentine junction/root surface (**EDJ/RS**) at Point **1** toward the pulpal dentine area (**PD**) at Point **10**. (The statistical analysis is carried out using the Mann-Whitney U test for the values in each point; **∗**p < 0.05; **∗∗**p < 0.01; **∗∗∗**p < 0.001)

The C values were highest close to the enamel-dentine junction/root surface (EDJ/RS) followed by a decrease to approximately the middle part of the dentine and then increased toward the pulpal dentine (PD) area. The C values were lower in DI-dentine and statistically significantly lower values were found in the three points closest to EDJ/RS.

The O values decreased from the EDJ/RS toward the PD, the values being higher in DI-dentine. In the points 4 and 7–9 the values were significantly higher in DI-dentine.

In normal dentine the Na values increased from the EDJ/RS toward the mid dentine and then decreased toward the PD. The Na profile in DI-dentine increased from the EDJ/RS toward the PD and had higher values compared with normal dentine. All values for Na in DI-dentine were significantly higher compared with normal dentine with the exception for point four.

In normal dentine the Mg concentration profile increased from the EDJ/RS toward the PD, which was in contrast to what was found in DI-dentine where the Mg decreased from the EDJ/RS. The Mg values in DI-dentine from point three and inwards were significantly lower compared with normal dentine.

In the P concentration profile the lowest values were found at the EDJ/RS with a rapid increase till point three and remained flat with a decrease close to the PD. There was no difference in the concentration profiles for P in normal and DI-dentine.

The Cl profile in normal and DI-dentine was at the same level and increased slightly toward the PD.

The K profile increased from the EDJ/RS toward the PD with approximately the same levels in normal and DI-dentine.

For Ca the concentration profiles were lowest at the EDJ/RS and increased till point four followed by slight decrease toward the PD. In the inner half of the dentine DI-dentine had lower values.

The concentration profile for the ratio Ca/P in normal and DI-dentine were parallel to each other. The ratio Ca/P had almost the same value in all measurement points with only a minor difference between normal and DI-dentine.

### Inductive analyses

The full results of the inductive analyses before and after 0.1% statistical pruning for the different elements are given in Table 3. The break point values were calculated by XpertRule Analyser.

**Table 3:** Results from the inductive analyses before pruning and after 0.1% statistical pruning of XRMA measurements in normal dentine and Dentinogenesis Imperfecta dentine showing the location (**Level**) in the induced knowledge tree for the elements **C, O, Na, Mg, P, Cl, K** and **Ca** and the outcome values normal dentine (**N**) and Dentinogenesis Imperfecta dentine (**DI**) with corresponding probability values within brackets

No pruning (number of end nodes = 26)			After pruning (number of end nodes = 11)		
Level	Element	Outcome (probability)	Element	Outcome (probability)	
1	Na		Na		
2	Na; Mg		Na; Mg		
3	C; O; Ca	DI (1.00)	C; O; Ca	DI (1.00)	
4	C; Mg	DI (1.00); DI (1.00)	Mg	DI (1.00); DI (1.00); N (0.98)	
5	C; O; Cl	DI (1.00); N (1.00); N (0.75); N (1.00)	C	DI (0.92); DI (1.00); N (1.00); N (0.92); N (0.64)	
6	C; Na	DI (0.75); DI (1.00); DI (0.71); N (1.00); N (1.00)		DI (0.80); N (0.74)	
7	O	DI (1.00); DI (0.75); N (1.00); N (1.00)			
8	C; O; Na; P				
9	Mg	DI (0.75); DI (1.00); DI (1.00); N (1.00); N (0.75); N (1.00); N (0.75)			
10	P	DI (1.00)			
11		DI (0.75); N (0.75)			

Redundant elements: K			P; Cl; K		
	% correctly classified			% correctly classified	
	Training	Test		Training	Test
Outcome results					
DI	98.4	94.6	DI	93.8	89.9
N	95.0	75.0	N	90.8	74.2
Overall	97.3	88.3	Overall	92.8	84.9

The outcome results are given for training set and the test set of data. (**Redundant element** = not appearing in the knowledge tree)

At the top level of booth knowledge trees was Na found and at the second level Na and Mg, thus being the two most important elements for discriminating normal dentine from DI-dentine. K did not appear in the knowledge tree, thus being redundant in the analysis. Before pruning the number of end nodes was 26 compared with 11 after statistical pruning of 0.1%. The percentage correctly classified outcome values “*DI-dentine*” and “*normal dentine*” before pruning were 98.4% and 95.0%, respectively. The corresponding values for the test set in which the induced rule was verified was lower (94.6% and 75.0%).

**Fig. 4:**
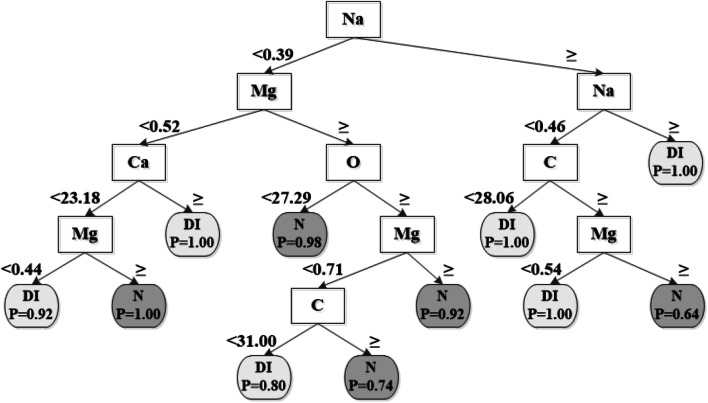
Knowledge tree induced in XpertRule Analyser after 0.1% statistical pruning using the XRMA values for **C, O, Na, Mg, P, Cl, K** and **Ca** in normal dentine and dentine in teeth from patients diagnosed with Dentinogenesis Imperfecta type II. Normal dentine (**N**) and dentine from Dentinogenesis Imperfecta type II (**DI**) were set as discrete outcome values in the analysis. The values at the straight arrows show the break points for respective element. In order to make the knowledge tree more feasible to read only the values of the left branch (**<**) are shown, the right branches having the same values after the sign **≥**. In the rounded outcome end nodes, the probability (**P=**) for correctly classified examples is given

In order to make the results more explicit only the knowledge tree after 0.1% statistical pruning is shown in Fig. 4. The first three levels of the knowledge tree contained the same elements before and after pruning. In the pruned knowledge tree P, Cl and K did not appear, thus being redundant. The percentage correctly classified outcome values “*DI-dentine*” and “*normal dentine*” after pruning were 93.8% and 90.8%, respectively. The corresponding values for the test set in which the induced rule was verified was lower (89.9% and 74.2%).

## Discussion

The present study has shown that the concentration profiles for C, O, Na and Mg measured by XRMA in dentine from primary teeth diagnosed with Dentinogenesis Imperfecta type II (DI) differ from normal primary dentine. The most marked differences were found for Na and Mg which was confirmed in the inductive analysis.

XRMA has proven to be a useful method for analysis of some inorganic elements in dental hard tissues with control of the morphological location of the measurements (Melin et al. 2014, 2015). Even if the values of the measurements are to be considered as semi-quantitative, comparisons between different locations and different samples can be made when the parameters for the XRMA analysis are well controlled.

Inductive analysis seen as a tool for pattern recognition is a complement to more traditional statistical methods and has been used in several areas of research (Nilsson et al. 1998; Klingberg et al. 1999; Melin et al. 2015). The evolved rules are induced using a randomised sample of 50% of the examples, these rules can then be evaluated in the verifying option of XpertRule Analyser against the remaining examples. The hierarchic presentation and the possibility for verifying the knowledge tree makes the inductive analysis a powerful tool in research.

The carbon values for DI-dentine were lower than found in normal dentine. In XRMA analysis carbon is a sum of what is located in the inorganic and organic matrix and may possibly reflect lower values of carbonates in the inorganic phase of the DI-dentine. Since the water content in DI-dentine is higher than in normal dentine is has been suggested that it may be related to water bound to the crystallite lattice in the inorganic phase, which could explain the increase of oxygen seen in DI-dentine (Kerebel et al. 1981).

The lower values of Mg in DI-dentine compared with normal dentine are in concordance with electron microprobe analyses of DI-dentine (Kerebel et al. 1981). It has been shown that in rat developing dentine that the ratio Mg/P from the peripheral dentine toward the central dentine increased (Steinfort et al. 1991). This in contrast to what was found in the present study with lower values of Mg decreasing from the enamel-dentine junction and inwards compared with normal dentine, possibly may be related to an impaired function of odontoblasts in the mineralization of dentine. In a study of bovine peritubular dentine it was shown that Mg^2+^ and K^+^ to a high extent are associated with the organic matrix, however, in the present study it was not possible to discriminate between peritubular and intertubular dentine (Gotliv et al. 2006).

It has previously been shown that an increase in the content of Mg may lead to a decrease in the Ca/P ratio, however, in the present study there were no differences found in the Ca/P ratio in DI-dentine and normal dentine (Stratman et al. 1996). The values for P, Ca and the ratio Ca/P did not differ between DI and normal dentine which is in contrast to what was found in a previous report (Kerebel et al. 1981). The relatively flat concentration profiles of P and Ca decreasing towards the pulpal dentine is in line with previous studies (Tøtdal and Hals 1985; Hals et al. 1988). The Ca/P ratio in the dentine in primary normal teeth and primary teeth from patients with hypophosphatemic rickets, showed only minor differences (Boukpessi et al. 2006). The ratio Ca/P is often presented in the literature as an indicator of the presence of hydroxyapatite in dental hard tissues. However, the Ca/P ratio is not regarded to be useful as an indicator of the calcium phosphate phase (Drouet 2013).

A previous study has shown that Na, Mg, P and Ca precipitated together in the formation of a homogenous carbonate apatite (Eidelman et al. 2009). In the present study the concentration profile of Na was inverted to what was found for Mg. The high values of Na are typical for extra cellular tissue in predentine and may reflect an impaired mineralization of DI-dentine (Wiesmann et al. 1995). In the inductive analysis Na was found at top node, thus being the most important element for discriminating DI-dentine from normal dentine, however, the underlying factors is at present not known.

## Conclusions

This study has shown that dentine in primary teeth from patients diagnosed with Dentinogenesis Imperfecta type II analysed with XRMA have lower values of C and Mg and higher values of O and Na compared with normal primary dentine. Further the inductive analysis revealed that Na was the most important element for discriminating DI-dentine from normal primary dentine.

## Electronic supplementary material

Below is the link to the electronic supplementary material.

Supplementary material 1 (DOCX 99 KB)
