# Supplementary material for: X-ray microanalysis of dentine in primary teeth diagnosed Dentinogenesis Imperfecta type II
Source: Eur Arch Paediatr Dent. 2019 Dec 10;21(4):527–35. doi: 10.1007/s40368-018-0392-2 (PMC7415746; doi:10.1007/s40368-018-0392-2)
Supplement: Supplementary file 1 — Supplementary material 1 (DOCX 99 KB) [file 40368_2018_392_MOESM1_ESM.docx]

Compiled data for carbon (**C**), oxygen (**O**), sodium (**Na**), magnesium (**Mg**), phosphorous (**P**), potassium (**K**) and calcium (**Ca**) in weight% from XRMA measurements in 5 primary teeth with normal dentine (**N**) and dentine in 7 primary teeth from patients diagnosed with Dentinogenesis Imperfecta type II (**DI**) for each sample (**Normal=N-01 – N-05; DI=DI-01 – DI-07**) in ten points (**Point 1-10**) from the enamel-dentine junction (**Point 1**) toward the pulpal dentine (**Point 10**). (**Mean**=mean value; **SD**=standard deviation.)

**Line A B C D Mean SD**

**N-01 Carbon**

**Point**

1 30.79 34.38 31.01 32.48 32.17 1.435

2 30.42 29.75 31.57 32.60 31.08 1.091

3 30.27 29.01 31.69 33.50 31.12 1.669

4 28.28 29.59 31.78 33.06 30.68 1.859

5 27.94 28.10 30.08 31.12 29.31 1.344

6 28.07 31.66 30.60 28.19 29.63 1.547

7 28.61 31.36 29.84 32.53 30.58 1.485

8 30.86 31.36 31.98 28.75 30.74 1.217

9 30.74 28.95 33.73 35.80 32.31 2.641

10 30.59 27.35 36.71 34.32 32.24 3.571

**N-01 Oxygen**

**Point**

1 30.43 32.52 32.55 33.42 32.23 1.100

2 34.15 29.11 28.92 29.83 30.50 2.131

3 32.51 28.39 26.96 28.74 29.15 2.053

4 30.15 27.71 27.20 27.14 28.05 1.233

5 30.56 31.95 29.32 29.16 30.25 1.122

6 30.72 26.17 27.94 29.69 28.63 1.733

7 27.82 29.94 32.41 28.38 29.64 1.777

8 31.05 29.49 28.83 26.53 28.98 1.625

9 26.12 29.34 25.49 20.46 25.35 3.183

10 29.92 28.94 27.82 28.14 28.71 0.811

**N-01 Sodium**

**Point**

1 0.32 0.28 0.35 0.21 0.29 0.052

2 0.26 0.42 0.35 0.28 0.33 0.063

3 0.29 0.43 0.40 0.30 0.36 0.060

4 0.38 0.39 0.42 0.37 0.39 0.019

5 0.52 0.40 0.44 0.40 0.44 0.048

6 0.45 0.35 0.37 0.32 0.37 0.046

7 0.44 0.37 0.35 0.33 0.37 0.039

8 0.33 0.38 0.31 0.25 0.32 0.046

9 0.35 0.38 0.23 0.20 0.29 0.079

10 0.51 0.37 0.20 0.27 0.34 0.117

**N-01 Magnesium**

**Point**

1 0.52 0.55 0.52 0.47 0.52 0.028

2 0.45 0.58 0.55 0.57 0.53 0.053

3 0.56 0.64 0.57 0.53 0.58 0.041

4 0.52 0.62 0.59 0.61 0.58 0.041

5 0.62 0.63 0.70 0.66 0.65 0.030

6 0.59 0.68 0.69 0.67 0.66 0.040

7 0.62 0.75 0.75 0.75 0.72 0.058

8 0.56 0.71 0.72 0.66 0.66 0.066

9 0.53 0.67 0.61 0.59 0.60 0.051

10 0.60 0.71 0.38 0.54 0.56 0.120

**Line A B C D Mean SD**

**N-01 Phosphorous**

**Point**

1 13.00 11.20 12.35 11.58 12.03 0.694

2 12.08 13.73 13.32 12.68 12.95 0.628

3 12.71 14.17 13.80 12.62 13.32 0.675

4 14.06 14.20 13.68 13.25 13.80 0.368

5 14.09 13.43 13.64 13.32 13.62 0.296

6 13.91 13.98 13.79 13.47 13.79 0.195

7 14.85 13.07 12.58 13.07 13.39 0.868

8 13.32 13.08 13.12 14.34 13.46 0.512

9 14.67 13.73 13.43 14.04 13.97 0.460

10 13.47 14.30 11.74 12.43 12.99 0.979

**N-01 Chlorine**

**Point**

1 0.09 0.13 0.13 0.18 0.13 0.033

2 0.08 0.08 0.07 0.08 0.08 0.005

3 0.06 0.07 0.07 0.07 0.07 0.006

4 0.05 0.08 0.07 0.07 0.07 0.009

5 0.05 0.05 0.07 0.06 0.06 0.006

6 0.05 0.07 0.08 0.05 0.06 0.013

7 0.07 0.06 0.06 0.06 0.06 0.003

8 0.05 0.06 0.06 0.06 0.06 0.005

9 0.07 0.09 0.05 0.06 0.07 0.015

10 0.07 0.06 0.03 0.03 0.05 0.017

**N-01 Potassium**

**Point**

1 0.01 0.02 0.00 0.02 0.01 0.006

2 0.01 0.00 0.01 0.01 0.01 0.003

3 0.02 0.00 0.01 0.01 0.01 0.006

4 0.01 0.01 0.01 0.01 0.01 0.003

5 0.01 0.01 0.02 0.01 0.01 0.004

6 0.02 0.00 0.02 0.02 0.02 0.007

7 0.02 0.01 0.01 0.02 0.02 0.004

8 0.01 0.02 0.01 0.01 0.01 0.004

9 0.02 0.02 0.02 0.02 0.02 0.003

10 0.02 0.02 0.01 0.01 0.02 0.004

**N-01 Calcium**

**Point**

1 24.84 20.92 23.09 21.63 22.62 1.500

2 22.56 26.33 25.21 23.95 24.51 1.404

3 23.59 27.29 26.50 24.24 25.40 1.535

4 26.55 27.40 26.25 25.50 26.42 0.681

5 26.21 25.42 25.74 25.28 25.66 0.358

6 26.19 27.09 26.52 27.58 26.84 0.532

7 27.57 24.44 24.00 24.85 25.22 1.393

8 23.81 24.90 24.97 29.40 25.77 2.146

9 27.49 26.80 26.44 28.84 27.39 0.916

10 24.81 28.25 23.10 24.25 25.10 1.916

**Line A B Mean SD**

**N-02 Carbon**

**Point**

1 34.84 35.19 35.01 0.171

2 29.80 33.12 31.46 1.661

3 30.65 32.15 31.40 0.750

4 31.71 31.00 31.36 0.357

5 30.93 29.68 30.30 0.626

6 29.86 31.26 30.56 0.700

7 31.28 31.93 31.60 0.327

8 31.91 30.90 31.41 0.503

9 31.07 32.26 31.66 0.594

10 39.52 31.87 35.70 3.824

**N-02 Oxygen**

**Point**

1 31.69 32.27 31.98 0.292

2 31.98 28.34 30.16 1.820

3 28.60 28.03 28.32 0.285

4 27.64 29.43 28.54 0.894

5 29.42 26.51 27.96 1.456

6 29.48 30.62 30.05 0.569

7 28.49 22.25 25.37 3.120

8 26.32 26.24 26.28 0.041

9 29.63 29.12 29.38 0.256

10 31.91 30.44 31.18 0.734

**N-02 Sodium**

**Point**

1 0.26 0.26 0.26 0.001

2 0.30 0.31 0.31 0.008

3 0.35 0.28 0.32 0.032

4 0.36 0.33 0.34 0.017

5 0.26 0.34 0.30 0.039

6 0.25 0.20 0.22 0.026

7 0.25 0.25 0.25 0.001

8 0.25 0.28 0.27 0.015

9 0.22 0.17 0.20 0.025

10 0.21 0.26 0.24 0.028

**N-02 Magnesium**

**Point**

1 0.60 0.64 0.62 0.017

2 0.58 0.65 0.61 0.031

3 0.67 0.64 0.66 0.014

4 0.71 0.73 0.72 0.012

5 0.74 0.66 0.70 0.038

6 0.77 0.70 0.74 0.035

7 0.78 0.73 0.76 0.024

8 0.78 0.79 0.79 0.004

9 0.75 0.71 0.73 0.024

10 0.57 0.74 0.66 0.086

**Line A B Mean SD**

**N-02 Phosphorous**

**Point**

1 11.53 11.24 11.39 0.148

2 13.23 13.00 13.12 0.115

3 13.75 13.46 13.60 0.144

4 13.72 13.48 13.60 0.117

5 13.55 14.55 14.05 0.504

6 13.86 13.27 13.57 0.298

7 13.65 14.97 14.31 0.660

8 14.01 14.39 14.20 0.189

9 13.41 13.02 13.22 0.195

10 9.83 12.71 11.27 1.443

**N-02 Chlorine**

**Point**

1 0.07 0.07 0.07 0.001

2 0.04 0.04 0.04 0.002

3 0.05 0.05 0.05 0.001

4 0.03 0.04 0.04 0.007

5 0.03 0.04 0.04 0.005

6 0.05 0.04 0.04 0.009

7 0.03 0.03 0.03 0.001

8 0.04 0.03 0.04 0.007

9 0.05 0.03 0.04 0.009

10 0.08 0.04 0.06 0.017

**N-02 Potassium**

**Point**

1 0.00 0.00 0.00 0.000

2 0.01 0.00 0.01 0.001

3 0.01 0.01 0.01 0.002

4 0.01 0.01 0.01 0.001

5 0.01 0.01 0.01 0.003

6 0.00 0.00 0.00 0.000

7 0.01 0.02 0.01 0.005

8 0.01 0.01 0.01 0.000

9 0.02 0.01 0.02 0.016

10 0.00 0.00 0.00 0.000

**N-02 Calcium**

**Point**

1 21.00 20.34 20.67 0.331

2 24.06 24.53 24.30 0.236

3 25.93 25.37 25.65 0.277

4 25.82 24.98 25.40 0.422

5 25.07 28.22 26.64 1.575

6 25.71 23.91 24.81 0.901

7 25.52 29.82 27.67 2.153

8 26.66 27.35 27.00 0.342

9 24.84 24.68 24.76 0.079

10 17.88 23.92 20.90 3.019

**Line A B C D Mean SD**

**N-03 Carbon**

**Point**

1 35.37 35.08 35.29 35.23 35.24 0.108

2 27.34 28.17 32.67 33.76 30.49 2.772

3 27.18 27.83 33.82 34.52 30.84 3.352

4 26.58 27.76 27.90 30.84 28.27 1.570

5 28.03 27.69 29.22 33.92 29.72 2.494

6 27.05 29.13 30.46 34.08 30.18 2.559

7 29.50 28.31 29.45 33.78 30.26 2.090

8 27.52 30.16 30.73 29.37 29.44 1.212

9 26.60 29.62 30.48 32.30 29.75 2.058

10 26.06 28.42 29.38 32.07 28.98 2.154

**N-03 Oxygen**

**Point**

1 26.75 26.23 25.49 23.75 25.55 1.134

2 29.91 28.98 23.26 25.88 27.01 2.631

3 30.00 30.46 26.95 25.02 28.11 2.233

4 31.57 29.86 26.92 27.15 28.88 1.938

5 30.87 29.03 28.20 24.81 28.22 2.196

6 31.43 26.82 26.91 25.60 27.69 2.218

7 29.52 27.92 27.76 24.00 27.30 2.027

8 30.90 27.05 26.60 27.37 27.98 1.710

9 31.02 28.53 25.76 23.78 27.27 2.743

10 32.30 28.27 26.16 19.40 26.53 4.673

**N-03 Sodium**

**Point**

1 0.26 0.29 0.20 0.25 0.25 0.030

2 0.28 0.37 0.28 0.24 0.29 0.049

3 0.33 0.31 0.22 0.27 0.28 0.041

4 0.33 0.28 0.31 0.29 0.30 0.018

5 0.30 0.29 0.33 0.29 0.30 0.017

6 0.27 0.29 0.28 0.27 0.28 0.011

7 0.27 0.27 0.27 0.25 0.26 0.008

8 0.27 0.29 0.24 0.25 0.26 0.022

9 0.25 0.26 0.26 0.17 0.23 0.038

10 0.23 0.22 0.20 0.10 0.19 0.053

**N-03 Magnesium**

**Point**

1 0.51 0.58 0.46 0.54 0.52 0.043

2 0.58 0.60 0.61 0.59 0.60 0.010

3 0.68 0.64 0.58 0.62 0.63 0.036

4 0.72 0.65 0.62 0.67 0.66 0.036

5 0.71 0.75 0.70 0.61 0.69 0.052

6 0.81 0.77 0.66 0.57 0.70 0.091

7 0.81 0.87 0.74 0.66 0.77 0.076

8 0.82 0.86 0.71 0.73 0.78 0.063

9 0.87 0.79 0.79 0.68 0.78 0.059

10 0.84 0.83 0.87 0.55 0.77 0.131

**Line A B C D Mean SD**

**N-03 Phosphorous**

**Point**

1 12.46 12.70 12.83 13.46 12.86 0.369

2 14.25 14.21 14.46 13.47 14.10 0.376

3 14.18 14.05 13.05 13.44 13.68 0.456

4 13.79 14.22 14.71 13.92 14.16 0.351

5 13.43 14.47 14.15 13.67 13.93 0.407

6 13.86 14.65 13.82 13.41 13.94 0.449

7 13.68 14.53 14.25 13.95 14.10 0.320

8 13.85 14.30 13.90 14.32 14.09 0.220

9 14.23 13.65 14.50 14.29 14.17 0.319

10 14.01 14.39 14.74 14.86 14.50 0.331

**N-03 Chlorine**

**Point**

1 0.03 0.04 0.05 0.04 0.04 0.010

2 0.04 0.03 0.03 0.03 0.03 0.003

3 0.04 0.04 0.04 0.03 0.04 0.005

4 0.03 0.05 0.03 0.04 0.04 0.007

5 0.03 0.03 0.04 0.03 0.03 0.005

6 0.02 0.04 0.03 0.03 0.03 0.006

7 0.03 0.03 0.04 0.04 0.04 0.005

8 0.03 0.03 0.05 0.03 0.04 0.010

9 0.02 0.03 0.03 0.05 0.03 0.009

10 0.04 0.04 0.03 0.07 0.05 0.016

**N-03 Potassium**

**Point**

1 0.00 0.01 0.00 0.00 0.00 0.003

2 0.00 0.01 0.00 0.01 0.01 0.003

3 0.00 0.00 0.00 0.00 0.00 0.001

4 0.01 0.01 0.00 0.01 0.01 0.003

5 0.00 0.00 0.01 0.00 0.00 0.003

6 0.01 0.01 0.00 0.01 0.01 0.004

7 0.00 0.01 0.02 0.00 0.01 0.007

8 0.02 0.00 0.01 0.01 0.01 0.006

9 0.00 0.00 0.01 0.01 0.01 0.004

10 0.00 0.00 0.01 0.01 0.01 0.005

**N-03 Calcium**

**Point**

1 24.64 25.08 25.67 26.73 25.53 0.785

2 27.59 27.63 28.69 26.03 27.48 0.949

3 27.60 26.68 25.33 26.09 26.43 0.832

4 26.97 27.18 29.51 27.09 27.69 1.056

5 26.63 27.74 27.35 26.67 27.10 0.468

6 26.55 28.29 27.86 26.02 27.18 0.927

7 26.20 28.06 27.48 27.32 27.27 0.676

8 26.59 27.32 27.76 27.91 27.40 0.513

9 27.00 27.14 28.18 28.73 27.76 0.722

10 26.53 27.84 28.61 32.94 28.98 2.407

**Line A B Mean SD**

**N-04 Carbon**

**Point**

1 34.92 34.25 34.59 0.332

2 31.99 32.21 32.10 0.108

3 29.30 29.44 29.37 0.070

4 32.75 28.20 30.47 2.275

5 32.04 26.53 29.28 2.757

6 31.31 30.88 31.09 0.215

7 33.26 31.71 32.48 0.779

8 33.88 31.11 32.50 1.381

9 34.55 35.05 34.80 0.250

10 32.14 31.89 32.01 0.128

**N-04 Oxygen**

**Point**

1 32.38 31.70 32.04 0.335

2 31.54 29.90 30.72 0.818

3 27.72 26.41 27.07 0.657

4 27.44 24.93 26.18 1.258

5 29.18 30.22 29.70 0.519

6 27.67 25.19 26.43 1.239

7 25.97 24.86 25.42 0.555

8 25.72 24.11 24.91 0.804

9 24.85 27.24 26.05 1.195

10 31.79 23.43 27.61 4.180

**N-04 Sodium**

**Point**

1 0.22 0.23 0.23 0.005

2 0.20 0.22 0.21 0.013

3 0.26 0.24 0.25 0.013

4 0.26 0.24 0.25 0.009

5 0.26 0.24 0.25 0.007

6 0.25 0.23 0.24 0.014

7 0.25 0.21 0.23 0.019

8 0.24 0.25 0.25 0.009

9 0.23 0.22 0.23 0.007

10 0.22 0.18 0.20 0.021

**N-04 Magnesium**

**Point**

1 0.53 0.54 0.54 0.006

2 0.49 0.53 0.51 0.020

3 0.56 0.57 0.56 0.009

4 0.62 0.66 0.64 0.020

5 0.68 0.63 0.65 0.023

6 0.73 0.68 0.70 0.025

7 0.77 0.70 0.73 0.037

8 0.76 0.84 0.80 0.039

9 0.78 0.77 0.77 0.005

10 0.85 0.76 0.80 0.045

**Line A B Mean SD**

**N-04 Phosphorous**

**Point**

1 11.29 11.67 11.48 0.193

2 12.57 12.84 12.70 0.132

3 14.32 14.53 14.42 0.110

4 13.50 14.94 14.22 0.719

5 13.14 14.23 13.69 0.546

6 13.80 14.36 14.08 0.281

7 13.63 14.14 13.89 0.253

8 13.48 14.97 14.22 0.746

9 13.66 12.64 13.15 0.508

10 12.45 14.65 13.55 1.101

**N-04 Chlorine**

**Point**

1 0.03 0.04 0.04 0.005

2 0.04 0.02 0.03 0.009

3 0.04 0.03 0.04 0.004

4 0.03 0.04 0.03 0.005

5 0.04 0.04 0.04 0.001

6 0.04 0.03 0.03 0.003

7 0.04 0.04 0.04 0.000

8 0.05 0.04 0.05 0.005

9 0.05 0.05 0.05 0.001

10 0.03 0.02 0.03 0.003

**N-04 Potassium**

**Point**

1 0.00 0.00 0.00 0.002

2 0.01 0.01 0.01 0.003

3 0.01 0.00 0.01 0.006

4 0.00 0.00 0.00 0.001

5 0.01 0.00 0.00 0.003

6 0.00 0.00 0.00 0.002

7 0.01 0.00 0.01 0.004

8 0.02 0.01 0.01 0.004

9 0.01 0.01 0.01 0.001

10 0.01 0.00 0.00 0.001

**N-04 Calcium**

**Point**

1 20.63 21.55 21.09 0.463

2 23.16 24.26 23.71 0.551

3 27.79 28.77 28.28 0.491

4 25.40 31.00 28.20 2.798

5 24.65 28.10 26.38 1.724

6 26.20 28.64 27.42 1.218

7 26.06 28.34 27.20 1.142

8 25.86 28.66 27.26 1.399

9 25.87 24.02 24.94 0.923

10 22.51 29.07 25.79 3.278

**Line A B C D Mean SD**

**N-05 Carbon**

**Point**

1 31.56 32.46 29.53 29.23 30.70 1.356

2 29.46 30.66 28.17 29.14 29.36 0.890

3 30.86 30.50 26.56 29.19 29.28 1.691

4 29.96 30.25 28.74 29.22 29.54 0.598

5 27.79 30.26 27.66 29.47 28.80 1.104

6 30.57 29.81 27.59 30.93 29.72 1.298

7 27.82 25.73 26.32 29.25 27.28 1.368

8 28.59 28.47 29.88 31.01 29.49 1.037

9 28.00 30.23 27.92 32.29 29.61 1.805

10 30.20 34.53 39.35 32.74 34.21 3.347

**N-05 Oxygen**

**Point**

1 24.98 29.61 23.14 25.88 25.90 2.356

2 30.65 26.70 27.82 25.18 27.59 2.003

3 28.67 29.78 28.86 28.11 28.86 0.602

4 26.02 27.60 30.28 26.52 27.60 1.646

5 22.53 30.11 30.36 27.29 27.57 3.150

6 27.93 31.28 29.44 25.97 28.65 1.951

7 25.00 34.12 27.58 24.56 27.82 3.815

8 25.38 31.29 23.33 26.20 26.55 2.931

9 29.99 27.14 24.55 23.51 26.30 2.507

10 27.19 22.67 24.29 26.48 25.16 1.792

**N-05 Sodium**

**Point**

1 0.22 0.21 0.19 0.28 0.23 0.035

2 0.25 0.34 0.26 0.29 0.28 0.035

3 0.20 0.26 0.25 0.24 0.24 0.022

4 0.18 0.35 0.25 0.25 0.26 0.058

5 0.32 0.24 0.23 0.24 0.26 0.036

6 0.21 0.26 0.30 0.23 0.25 0.036

7 0.24 0.26 0.25 0.22 0.24 0.016

8 0.28 0.22 0.19 0.23 0.23 0.032

9 0.31 0.21 0.14 0.13 0.20 0.073

10 0.38 0.11 0.14 0.20 0.21 0.106

**N-05 Magnesium**

**Point**

1 0.60 0.56 0.43 0.87 0.62 0.161

2 0.53 0.63 0.63 0.64 0.61 0.048

3 0.56 0.64 0.63 0.66 0.62 0.038

4 0.60 0.71 0.72 0.67 0.67 0.047

5 0.70 0.73 0.68 0.65 0.69 0.027

6 0.72 0.74 0.79 0.68 0.73 0.038

7 0.78 0.75 0.75 0.69 0.74 0.030

8 0.80 0.78 0.75 0.86 0.80 0.041

9 0.90 0.74 0.82 0.84 0.82 0.059

10 0.69 0.55 0.52 0.63 0.60 0.066

**Line A B C D Mean SD**

**N-05 Phosphorous**

**Point**

1 14.67 12.81 14.83 14.33 14.16 0.801

2 13.70 14.18 14.36 14.74 14.24 0.376

3 14.09 13.57 15.04 14.36 14.26 0.529

4 15.37 14.21 13.81 14.59 14.50 0.577

5 16.28 13.48 14.17 14.31 14.56 1.039

6 14.30 13.23 14.35 14.35 14.06 0.476

7 15.45 13.33 14.92 14.70 14.60 0.781

8 15.28 13.76 15.02 14.13 14.55 0.623

9 14.25 14.43 15.67 14.45 14.70 0.563

10 14.29 13.18 12.06 13.30 13.21 0.792

**N-05 Chlorine**

**Point**

1 0.08 0.09 0.19 0.05 0.10 0.051

2 0.04 0.05 0.06 0.04 0.05 0.008

3 0.04 0.04 0.04 0.04 0.04 0.004

4 0.04 0.05 0.04 0.03 0.04 0.009

5 0.08 0.05 0.04 0.03 0.05 0.017

6 0.02 0.05 0.04 0.03 0.04 0.011

7 0.04 0.05 0.05 0.04 0.04 0.003

8 0.03 0.05 0.04 0.04 0.04 0.005

9 0.06 0.04 0.10 0.04 0.06 0.024

10 0.08 0.27 0.24 0.03 0.15 0.099

**N-05 Potassium**

**Point**

1 0.01 0.00 0.01 0.02 0.01 0.006

2 0.01 0.01 0.00 0.02 0.01 0.007

3 0.02 0.01 0.02 0.01 0.01 0.004

4 0.03 0.00 0.01 0.02 0.01 0.012

5 0.03 0.00 0.01 0.02 0.02 0.011

6 0.01 0.00 0.01 0.00 0.01 0.004

7 0.02 0.01 0.01 0.03 0.02 0.006

8 0.02 0.01 0.01 0.01 0.01 0.002

9 0.02 0.01 0.01 0.02 0.01 0.004

10 -0.01 0.01 0.01 0.03 0.01 0.015

**N-05 Calcium**

**Point**

1 27.87 24.25 31.67 29.33 28.28 2.694

2 25.36 27.43 28.70 29.95 27.86 1.694

3 25.56 25.20 28.61 27.39 26.69 1.385

4 27.79 26.84 26.16 28.71 27.37 0.964

5 32.28 25.13 26.85 27.99 28.06 2.640

6 26.25 24.62 27.49 27.80 26.54 1.250

7 30.65 25.76 30.12 30.51 29.26 2.029

8 29.62 25.42 30.78 27.52 28.33 2.048

9 26.47 27.20 30.79 28.71 28.29 1.652

10 27.18 28.68 23.40 26.59 26.46 1.924

**Line A B C D Mean SD**

**DI-01 Carbon**

**Point**

1 28.44 27.11 28.07 27.20 27.70 0.567

2 28.95 26.66 26.27 27.07 27.24 1.029

3 29.20 28.48 30.97 28.03 29.17 1.121

4 27.73 28.79 29.26 28.25 28.51 0.576

5 27.76 29.53 26.47 29.75 28.38 1.344

6 28.71 29.45 29.21 28.45 28.95 0.394

7 29.75 29.35 31.35 30.11 30.14 0.748

8 31.70 30.56 28.61 27.56 29.61 1.616

9 30.45 30.67 27.20 31.47 29.95 1.631

10 33.35 26.89 27.00 29.38 29.15 2.619

**DI-01 Oxygen**

**Point**

1 30.80 31.39 28.43 28.91 29.88 1.242

2 32.73 30.01 31.70 27.78 30.55 1.874

3 28.13 27.17 29.27 27.46 28.01 0.806

4 27.65 25.75 30.75 30.81 28.74 2.146

5 27.48 30.58 27.96 30.10 29.03 1.335

6 28.36 27.58 28.73 25.21 27.47 1.367

7 23.72 30.97 30.33 31.08 29.03 3.079

8 24.18 30.87 28.55 25.76 27.34 2.569

9 22.74 25.12 26.84 26.11 25.20 1.547

10 24.93 28.60 28.38 23.59 26.37 2.167

**DI-01 Sodium**

**Point**

1 0.31 0.44 0.46 0.36 0.39 0.058

2 0.26 0.35 0.32 0.40 0.33 0.051

3 0.34 0.45 0.33 0.44 0.39 0.057

4 0.30 0.51 0.28 0.25 0.33 0.103

5 0.34 0.34 0.44 0.29 0.35 0.053

6 0.32 0.39 0.35 0.41 0.37 0.036

7 0.30 0.33 0.28 0.29 0.30 0.018

8 0.29 0.29 0.34 0.41 0.33 0.050

9 0.28 0.39 0.43 0.37 0.37 0.052

10 0.38 0.40 0.50 0.53 0.45 0.063

**DI-01 Magnesium**

**Point**

1 0.71 0.71 0.76 0.68 0.72 0.029

2 0.63 0.70 0.65 0.66 0.66 0.023

3 0.57 0.51 0.55 0.64 0.57 0.047

4 0.59 0.55 0.63 0.64 0.60 0.036

5 0.57 0.41 0.54 0.66 0.55 0.089

6 0.58 0.53 0.55 0.73 0.60 0.080

7 0.29 0.58 0.60 0.55 0.51 0.126

8 0.25 0.58 0.60 0.54 0.49 0.139

9 0.24 0.60 0.57 0.45 0.47 0.143

10 0.24 0.68 0.59 0.46 0.49 0.163

**Line A B C D Mean SD**

**DI-01 Phosphorous**

**Point**

1 13.63 13.66 14.25 14.59 14.03 0.407

2 13.00 14.59 14.15 15.06 14.20 0.761

3 14.42 14.78 13.28 14.58 14.26 0.581

4 14.81 15.21 13.60 13.78 14.35 0.678

5 15.02 13.64 14.97 13.61 14.31 0.684

6 14.62 14.46 14.09 15.26 14.61 0.421

7 14.81 13.56 12.85 13.09 13.57 0.755

8 14.25 13.03 14.33 15.26 14.22 0.793

9 14.97 14.58 14.99 13.89 14.61 0.443

10 13.56 14.72 14.63 15.25 14.54 0.614

**DI-01 Chlorine**

**Point**

1 0.13 0.08 0.10 0.06 0.09 0.028

2 0.06 0.04 0.04 0.04 0.05 0.007

3 0.04 0.04 0.04 0.04 0.04 0.003

4 0.05 0.05 0.05 0.04 0.04 0.005

5 0.05 0.05 0.06 0.04 0.05 0.004

6 0.05 0.06 0.05 0.04 0.05 0.009

7 0.05 0.04 0.03 0.04 0.04 0.008

8 0.08 0.05 0.05 0.06 0.06 0.012

9 0.14 0.06 0.03 0.05 0.07 0.039

10 0.10 0.06 0.06 0.05 0.07 0.021

**DI-01 Potassium**

**Point**

1 0.03 0.01 0.03 0.01 0.02 0.008

2 0.00 0.00 0.00 0.01 0.00 0.003

3 0.00 0.01 0.00 0.00 0.00 0.004

4 0.01 0.01 0.01 0.00 0.01 0.004

5 0.00 0.01 0.01 0.00 0.01 0.006

6 0.01 0.01 0.01 0.01 0.01 0.003

7 0.01 0.00 0.02 0.01 0.01 0.005

8 0.00 0.01 0.00 0.01 0.00 0.003

9 0.01 0.01 0.00 0.00 0.01 0.006

10 0.01 0.00 0.00 0.01 0.00 0.003

**DI-01 Calcium**

**Point**

1 25.95 26.60 27.90 28.18 27.70 0.567

2 24.37 27.68 26.86 28.99 27.24 1.029

3 27.30 28.57 25.57 28.80 29.17 1.121

4 28.87 29.12 25.42 26.25 28.51 0.576

5 28.78 25.43 29.56 25.53 28.38 1.344

6 27.37 27.53 27.00 29.89 28.95 0.394

7 31.07 25.17 24.54 24.84 30.14 0.748

8 29.26 24.63 27.53 30.41 29.61 1.616

9 31.16 28.57 29.95 27.67 29.95 1.631

10 27.43 28.66 28.84 30.74 29.15 2.619

**Line A B C D E F Mean SD**

**DI-02 Carbon**

**Point**

1 29.49 33.78 38.13 28.25 28.73 29.32 31.28 3.556

2 27.28 29.73 35.72 26.80 27.21 29.90 29.44 3.064

3 27.44 30.92 31.80 26.69 27.89 27.79 28.76 1.900

4 29.60 28.95 32.25 26.72 27.77 28.03 28.89 1.756

5 32.05 27.78 30.44 26.88 27.78 27.40 28.72 1.869

6 30.63 30.44 32.33 27.07 27.92 28.47 29.48 1.809

7 30.64 30.05 30.65 27.75 28.89 29.30 29.55 1.033

8 30.12 30.49 29.58 33.29 28.05 28.83 30.06 1.653

9 32.24 29.60 29.87 30.58 31.32 28.26 30.31 1.272

10 29.73 30.68 30.59 30.53 30.53 29.64 30.28 0.425

**DI-02 Oxygen**

**Point**

1 33.33 31.56 29.02 30.71 30.17 32.58 31.23 1.450

2 31.51 34.09 32.07 32.43 30.22 27.04 31.23 2.199

3 33.15 34.63 29.44 30.59 30.24 28.23 31.05 2.185

4 31.33 32.24 32.07 30.77 37.10 30.28 32.30 2.252

5 32.39 31.27 30.60 29.77 29.50 29.20 30.45 1.111

6 34.01 33.69 31.83 28.26 26.53 29.50 30.64 2.767

7 34.88 29.79 35.18 28.26 29.53 28.84 31.08 2.837

8 32.49 34.08 29.07 27.68 31.76 30.58 30.94 2.130

9 33.52 30.45 33.10 28.62 28.18 30.99 30.81 2.020

10 30.97 28.94 28.79 30.23 28.68 29.72 29.55 0.839

**DI-02 Sodium**

**Point**

1 0.46 0.34 0.31 0.40 0.45 0.39 0.39 0.053

2 0.45 0.33 0.28 0.35 0.42 0.40 0.37 0.057

3 0.36 0.30 0.32 0.34 0.37 0.41 0.35 0.036

4 0.35 0.35 0.29 0.34 0.30 0.35 0.33 0.026

5 0.30 0.36 0.32 0.31 0.38 0.38 0.34 0.035

6 0.30 0.30 0.27 0.30 0.41 0.39 0.33 0.053

7 0.27 0.27 0.27 0.54 0.45 0.45 0.37 0.110

8 0.33 0.28 0.43 0.48 0.40 0.33 0.37 0.068

9 0.36 0.52 0.33 0.54 0.50 0.36 0.44 0.086

10 0.53 0.57 0.54 0.51 0.55 0.47 0.53 0.032

**DI-02 Magnesium**

**Point**

1 0.75 0.30 0.29 0.69 0.49 0.48 0.50 0.174

2 0.59 0.71 0.54 0.69 0.59 0.49 0.60 0.076

3 0.47 0.69 0.60 0.69 0.54 0.51 0.58 0.084

4 0.37 0.68 0.63 0.63 0.50 0.46 0.55 0.112

5 0.37 0.66 0.61 0.60 0.47 0.46 0.53 0.101

6 0.54 0.65 0.60 0.53 0.45 0.39 0.53 0.086

7 0.52 0.54 0.58 0.56 0.45 0.39 0.51 0.066

8 0.61 0.56 0.60 0.48 0.47 0.38 0.52 0.080

9 0.58 0.61 0.49 0.50 0.50 0.38 0.51 0.073

10 0.65 0.59 0.56 0.48 0.51 0.39 0.53 0.082

**Line A B C D E F Mean SD**

**DI-02 Phosphorous**

**Point**

1 12.16 11.12 10.50 13.38 13.14 12.25 12.09 1.022

2 13.78 12.17 10.72 13.50 13.86 13.66 12.95 1.147

3 13.46 11.76 12.75 13.96 13.58 14.20 13.28 0.819

4 13.01 13.13 11.98 13.87 11.83 13.49 12.89 0.746

5 11.86 13.16 12.85 14.06 13.68 13.99 13.27 0.762

6 12.12 12.21 12.12 14.19 14.28 13.39 13.05 0.945

7 12.28 13.93 11.94 14.07 13.22 13.43 13.14 0.792

8 12.68 11.98 13.51 12.64 13.05 13.18 12.84 0.487

9 11.39 12.92 12.61 13.27 12.99 13.14 12.72 0.630

10 12.81 13.10 13.25 12.78 13.20 13.12 13.04 0.183

**DI-02 Chlorine**

**Point**

1 0.06 0.05 0.03 0.03 0.03 0.03 0.04 0.012

2 0.11 0.04 0.08 0.04 0.05 0.04 0.06 0.027

3 0.05 0.04 0.06 0.05 0.03 0.05 0.05 0.008

4 0.06 0.04 0.04 0.05 0.04 0.04 0.05 0.007

5 0.05 0.05 0.06 0.04 0.04 0.04 0.05 0.006

6 0.04 0.04 0.04 0.06 0.04 0.05 0.05 0.008

7 0.07 0.04 0.05 0.04 0.04 0.05 0.05 0.009

8 0.06 0.04 0.05 0.04 0.06 0.04 0.05 0.009

9 0.04 0.05 0.05 0.03 0.02 0.05 0.04 0.011

10 0.04 0.04 0.06 0.05 0.02 0.05 0.04 0.011

**DI-02 Potassium**

**Point**

1 0.02 0.02 0.01 0.00 0.01 0.01 0.01 0.005

2 0.02 0.00 0.01 0.00 0.01 0.01 0.01 0.006

3 0.03 0.00 0.00 0.00 0.01 0.01 0.01 0.009

4 0.01 0.00 0.00 0.00 0.01 0.01 0.00 0.003

5 0.01 0.00 0.00 0.00 0.01 0.00 0.00 0.004

6 0.01 0.02 0.00 0.00 0.02 0.01 0.01 0.008

7 0.00 0.01 0.01 0.00 0.00 0.00 0.00 0.002

8 0.01 0.01 0.01 0.01 0.00 0.02 0.01 0.005

9 0.00 0.00 0.00 0.02 0.00 0.00 0.00 0.006

10 0.01 0.00 0.02 0.00 0.00 0.00 0.01 0.006

**DI-02 Calcium**

**Point**

1 23.73 22.83 21.70 26.53 26.97 24.93 24.45 1.899

2 26.26 22.93 20.57 26.19 27.64 28.46 25.34 2.744

3 25.03 21.66 25.04 27.69 27.34 28.80 25.93 2.349

4 25.27 24.62 22.73 27.60 22.45 27.34 25.00 2.006

5 22.97 26.72 25.13 28.35 28.14 28.52 26.64 2.021

6 22.35 22.64 22.81 29.58 30.35 27.80 25.92 3.407

7 21.36 25.38 21.31 28.77 27.41 27.54 25.29 2.92

8 23.71 22.56 26.74 25.38 26.21 26.64 25.21 1.564

9 21.88 25.84 23.55 26.43 26.49 26.81 25.17 1.822

10 25.25 26.09 26.20 25.42 26.51 26.62 26.02 0.515

**Line A B C D Mean SD**

**DI-03 Carbon** (MV=missing value)

**Point**

1 30.39 34.97 34.32 37.15 34.21 2.441

2 27.65 27.45 32.71 31.97 29.94 2.413

3 27.48 27.44 30.66 32.01 29.40 1.997

4 29.51 26.30 31.57 34.34 30.43 2.939

5 29.41 26.65 30.53 36.46 30.76 3.578

6 26.11 26.12 35.59 36.58 31.10 4.998

7 25.11 28.80 33.63 36.54 31.02 4.394

8 26.02 25.65 34.11 34.58 30.09 4.260

9 24.40 26.72 34.83 34.27 30.05 4.573

10 MV 32.33 37.40 35.37 35.03 2.086

**DI-03 Oxygen** (MV=missing value)

**Point**

1 31.94 25.07 28.54 25.54 27.77 2.749

2 27.94 28.33 23.17 24.02 25.86 2.292

3 27.63 26.14 25.38 23.83 25.74 1.371

4 25.77 28.32 27.47 27.73 27.33 0.948

5 28.36 28.55 27.32 27.59 27.95 0.512

6 29.20 29.12 26.14 27.60 28.02 1.257

7 32.02 25.51 25.25 28.36 27.78 2.734

8 28.08 27.32 20.61 29.92 26.48 3.520

9 31.52 29.22 22.82 28.97 28.13 3.225

10 MV 26.28 29.44 25.83 27.18 1.604

**DI-03 Sodium** (MV=missing value)

**Point**

1 0.30 0.25 0.22 0.22 0.25 0.032

2 0.25 0.27 0.17 0.18 0.22 0.043

3 0.24 0.29 0.21 0.18 0.23 0.041

4 0.26 0.23 0.25 0.15 0.22 0.043

5 0.20 0.27 0.24 0.17 0.22 0.039

6 0.23 0.22 0.18 0.21 0.21 0.018

7 0.22 0.29 0.23 0.19 0.23 0.033

8 0.21 0.24 0.22 0.16 0.21 0.030

9 0.27 0.27 0.19 0.18 0.23 0.042

10 MV 0.26 0.18 0.20 0.21 0.035

**DI-03 Magnesium** (MV=missing value)

**Point**

1 0.47 0.46 0.45 0.40 0.45 0.027

2 0.47 0.48 0.44 0.38 0.44 0.038

3 0.45 0.52 0.48 0.39 0.46 0.048

4 0.45 0.53 0.49 0.37 0.46 0.060

5 0.46 0.54 0.53 0.41 0.48 0.053

6 0.47 0.55 0.49 0.43 0.48 0.043

7 0.50 0.48 0.50 0.39 0.47 0.045

8 0.47 0.53 0.48 0.43 0.48 0.037

9 0.52 0.49 0.48 0.43 0.48 0.031

10 MV 0.46 0.40 0.43 0.43 0.023

**Line A B C D Mean SD**

**DI-03 Phosphorous** (MV=missing value)

**Point**

1 12.71 13.28 12.43 12.05 12.62 0.448

2 14.61 14.51 14.52 14.05 14.42 0.220

3 14.79 15.34 14.57 13.98 14.67 0.488

4 14.73 15.08 13.69 12.90 14.10 0.863

5 14.21 15.09 14.12 12.23 13.91 1.041

6 14.88 15.04 13.03 12.16 13.78 1.225

7 14.62 14.82 13.81 11.80 13.76 1.197

8 15.25 15.75 14.87 12.15 14.50 1.395

9 14.84 14.80 14.14 12.47 14.06 0.963

10 MV 13.66 11.20 13.05 12.64 1.049

**DI-03 Chlorine** (MV=missing value)

**Point**

1 0.04 0.05 0.05 0.05 0.05 0.003

2 0.05 0.05 0.06 0.05 0.05 0.004

3 0.05 0.06 0.06 0.06 0.06 0.007

4 0.05 0.05 0.05 0.04 0.05 0.006

5 0.06 0.05 0.06 0.04 0.05 0.009

6 0.06 0.05 0.05 0.05 0.05 0.004

7 0.06 0.06 0.04 0.04 0.05 0.008

8 0.07 0.06 0.06 0.03 0.06 0.013

9 0.08 0.05 0.05 0.02 0.05 0.020

10 MV 0.13 0.06 0.05 0.08 0.037

**DI-03 Potassium** (MV=missing value)

**Point**

1 0.00 0.00 0.00 0.01 0.00 0.002

2 0.00 0.01 0.00 0.00 0.00 0.006

3 0.01 0.01 0.00 0.00 0.00 0.003

4 0.00 0.00 0.01 0.00 0.00 0.003

5 0.00 0.00 0.00 0.00 0.00 0.001

6 0.00 0.00 0.01 0.00 0.00 0.005

7 0.00 0.00 0.00 0.00 0.00 0.002

8 0.01 0.00 0.00 0.01 0.00 0.004

9 0.00 0.00 0.00 0.00 0.00 0.000

10 MV 0.00 0.00 0.00 0.00 0.002

**DI-03 Calcium** (MV=missing value)

**Point**

1 24.16 25.92 24.00 24.59 24.67 0.754

2 29.05 28.91 28.92 29.38 29.06 0.189

3 29.36 30.19 28.64 29.56 29.44 0.554

4 29.24 29.49 26.46 24.47 27.42 2.076

5 27.29 28.86 27.21 23.10 26.61 2.134

6 29.06 28.91 24.51 22.99 26.37 2.673

7 27.47 30.05 26.55 22.67 26.68 2.650

8 29.91 30.44 29.64 22.71 28.18 3.166

9 28.38 28.45 27.50 23.67 27.00 1.958

10 MV 26.88 21.32 25.07 24.42 2.313

**Line A B C D E Mean SD**

**DI-04 Carbon**

**Point**

1 34.01 31.65 31.61 31.77 35.63 32.94 1.622

2 30.29 29.38 29.48 31.59 30.50 30.25 0.800

3 28.87 27.91 29.88 26.25 28.82 28.34 1.220

4 27.27 31.30 30.20 30.61 30.27 29.93 1.387

5 27.93 28.11 29.19 30.10 31.05 29.27 1.185

6 26.93 29.44 32.51 30.18 32.63 30.34 2.116

7 29.37 29.65 30.02 32.78 33.46 31.06 1.709

8 31.71 31.04 35.00 32.49 30.99 32.25 1.479

9 30.95 31.04 36.12 35.43 32.06 33.12 2.214

10 30.85 35.02 35.26 33.66 32.06 33.37 1.698

**DI-04 Oxygen**

**Point**

1 29.91 31.91 30.96 30.33 29.60 30.54 0.824

2 25.32 28.94 26.09 28.70 28.93 27.59 1.565

3 26.70 28.51 25.50 31.36 31.97 28.81 2.530

4 29.02 31.46 25.31 27.57 23.20 27.31 2.865

5 29.81 28.07 27.81 26.99 23.21 27.18 2.186

6 28.79 28.79 30.52 29.11 27.43 28.93 0.985

7 30.68 29.83 28.93 26.55 29.29 29.06 1.386

8 30.28 25.82 28.09 26.45 29.82 28.09 1.766

9 29.40 27.30 25.79 28.35 27.13 27.59 1.215

10 31.67 28.50 26.09 26.13 26.16 27.71 2.183

**DI-04 Sodium**

**Point**

1 0.36 0.42 0.36 0.29 0.50 0.39 0.069

2 0.45 0.35 0.47 0.30 0.36 0.38 0.062

3 0.45 0.36 0.41 0.82 0.37 0.48 0.174

4 0.68 0.27 0.51 0.48 0.40 0.47 0.135

5 0.62 0.36 0.65 0.71 0.65 0.60 0.122

6 0.80 0.43 0.45 0.65 0.57 0.58 0.136

7 0.80 0.52 0.91 0.69 0.60 0.71 0.140

8 0.56 0.75 0.59 0.92 0.70 0.70 0.129

9 0.67 0.84 0.53 0.53 0.70 0.65 0.119

10 0.55 0.57 0.86 0.79 0.72 0.70 0.119

**DI-04 Magnesium**

**Point**

1 0.54 0.49 0.52 0.55 0.65 0.55 0.056

2 0.44 0.49 0.61 0.65 0.69 0.57 0.096

3 0.43 0.48 0.49 1.12 0.93 0.69 0.281

4 0.66 0.48 0.49 0.60 0.68 0.58 0.081

5 0.62 0.59 0.57 0.60 0.73 0.62 0.057

6 0.59 0.59 0.50 0.60 0.59 0.57 0.037

7 0.65 0.60 0.72 0.54 0.70 0.64 0.065

8 0.63 0.55 0.50 0.85 0.77 0.66 0.132

9 0.49 0.63 0.55 0.66 0.70 0.61 0.075

10 0.52 0.47 0.55 0.57 0.59 0.54 0.040

**Line A B C D E Mean SD**

**DI-04 Phosphorous**

**Point**

1 12.52 12.54 13.21 13.19 12.34 12.76 0.364

2 14.76 14.59 15.22 13.73 13.87 14.43 0.559

3 14.96 14.81 15.14 15.02 14.32 14.85 0.286

4 15.42 13.19 15.19 14.47 15.56 14.77 0.875

5 15.42 14.93 14.78 15.04 15.57 15.15 0.298

6 15.53 14.85 13.50 14.87 14.74 14.70 0.660

7 14.26 15.03 14.81 14.60 14.01 14.54 0.368

8 14.04 14.93 13.58 15.13 14.69 14.47 0.577

9 14.13 15.03 13.73 13.57 14.85 14.26 0.585

10 13.69 13.11 13.83 14.47 15.11 14.04 0.689

**DI-04 Chlorine**

**Point**

1 0.05 0.06 0.07 0.12 0.05 0.07 0.024

2 0.07 0.06 0.06 0.08 0.06 0.07 0.007

3 0.05 0.06 0.07 0.05 0.04 0.05 0.008

4 0.05 0.06 0.04 0.05 0.05 0.05 0.008

5 0.05 0.06 0.05 0.04 0.05 0.05 0.006

6 0.04 0.05 0.05 0.05 0.05 0.05 0.006

7 0.05 0.04 0.06 0.06 0.06 0.05 0.007

8 0.04 0.04 0.05 0.04 0.04 0.04 0.004

9 0.06 0.05 0.03 0.04 0.05 0.05 0.012

10 0.06 0.05 0.04 0.04 0.05 0.05 0.008

**DI-04 Potassium**

**Point**

1 0.00 0.00 0.00 0.00 0.02 0.01 0.009

2 0.01 0.00 0.02 0.00 0.00 0.01 0.006

3 0.00 0.01 0.00 0.00 0.01 0.01 0.005

4 0.02 0.01 0.02 0.01 0.01 0.01 0.006

5 0.02 0.00 0.03 0.03 0.01 0.02 0.010

6 0.03 0.01 0.00 0.01 0.02 0.02 0.009

7 0.01 0.02 0.00 0.01 0.02 0.01 0.006

8 0.02 0.02 0.02 0.02 0.01 0.02 0.006

9 0.01 0.01 0.03 0.01 0.01 0.01 0.006

10 0.01 0.02 0.02 0.03 0.04 0.02 0.008

**DI-04 Calcium**

**Point**

1 22.60 22.93 23.27 23.77 21.20 22.75 0.867

2 28.67 26.18 28.07 24.96 25.59 26.70 1.434

3 28.53 27.87 28.51 25.36 23.53 26.76 1.991

4 26.88 23.23 28.23 26.21 29.82 26.87 2.203

5 25.54 27.88 26.92 26.49 28.74 27.12 1.106

6 27.30 25.84 22.47 24.53 23.98 24.82 1.645

7 24.17 24.31 24.54 24.77 21.87 23.93 1.049

8 22.74 26.84 22.18 24.10 22.99 23.77 1.659

9 24.29 25.10 23.23 21.41 24.49 23.70 1.294

10 22.64 22.25 23.36 24.32 25.28 23.57 1.108

**Line A B Mean SD**

**DI-05 Carbon**

**Point**

1 27.57 22.94 25.25 2.318

2 25.37 21.57 23.47 1.902

3 24.66 22.73 23.70 0.967

4 24.14 22.14 23.14 0.998

5 23.43 22.17 22.80 0.628

6 24.61 23.95 24.28 0.329

7 24.82 24.74 24.78 0.041

8 24.73 24.02 24.37 0.353

9 23.61 24.67 24.14 0.533

10 25.45 24.91 25.18 0.266

**DI-05 Oxygen**

**Point**

1 32.04 39.19 35.61 3.576

2 33.48 40.20 36.84 3.364

3 29.39 32.90 31.14 1.757

4 30.37 37.99 34.18 3.809

5 35.64 37.15 36.39 0.757

6 33.43 33.32 33.37 0.055

7 31.76 31.08 31.42 0.337

8 33.03 31.09 32.06 0.971

9 33.94 27.91 30.93 3.013

10 35.21 28.14 31.68 3.533

**DI-05 Sodium**

**Point**

1 0.48 0.41 0.44 0.031

2 0.45 0.39 0.42 0.031

3 0.43 0.50 0.47 0.038

4 0.40 0.67 0.53 0.137

5 0.43 0.40 0.41 0.015

6 0.52 0.43 0.48 0.043

7 0.47 0.61 0.54 0.068

8 0.52 0.38 0.45 0.068

9 0.51 0.38 0.45 0.065

10 0.64 0.45 0.54 0.098

**DI-05 Magnesium**

**Point**

1 0.58 0.56 0.57 0.005

2 0.51 0.53 0.52 0.010

3 0.48 0.51 0.50 0.015

4 0.50 0.44 0.47 0.033

5 0.45 0.50 0.47 0.023

6 0.44 0.56 0.50 0.056

7 0.44 0.44 0.44 0.002

8 0.52 0.43 0.48 0.041

9 0.46 0.46 0.46 0.000

10 0.51 0.37 0.44 0.073

**Line A B Mean SD**

**DI-05 Phosphorous**

**Point**

1 12.91 12.27 12.59 0.323

2 13.16 12.88 13.02 0.140

3 14.29 14.51 14.40 0.107

4 14.40 13.32 13.86 0.540

5 13.41 13.49 13.45 0.036

6 13.63 13.98 13.81 0.176

7 14.03 14.30 14.17 0.132

8 13.97 14.56 14.27 0.295

9 14.13 15.19 14.66 0.527

10 13.15 15.15 14.15 1.002

**DI-05 Chlorine**

**Point**

1 0.03 0.02 0.03 0.005

2 0.03 0.02 0.03 0.006

3 0.03 0.04 0.04 0.003

4 0.04 0.01 0.03 0.015

5 0.04 0.03 0.03 0.006

6 0.03 0.04 0.03 0.008

7 0.05 0.04 0.04 0.006

8 0.04 0.05 0.05 0.003

9 0.04 0.05 0.04 0.007

10 0.05 0.05 0.05 0.001

**DI-05 Potassium**

**Point**

1 0.01 0.01 0.01 0.003

2 0.00 0.01 0.01 0.002

3 0.02 0.01 0.01 0.005

4 0.01 0.01 0.01 0.000

5 0.02 0.01 0.02 0.002

6 0.00 0.01 0.01 0.006

7 0.01 0.02 0.01 0.006

8 0.01 0.02 0.01 0.003

9 0.01 0.02 0.01 0.004

10 0.00 0.01 0.01 0.004

**DI-05 Calcium**

**Point**

1 26.38 24.60 25.49 0.890

2 27.00 24.40 25.70 1.298

3 30.70 28.80 29.75 0.947

4 30.14 25.42 27.78 2.360

5 26.59 26.25 26.42 0.166

6 27.34 27.70 27.52 0.182

7 28.42 28.78 28.60 0.181

8 27.18 29.44 28.31 1.131

9 27.31 31.32 29.31 2.006

10 24.99 30.91 27.95 2.962

**Line A B C Mean SD**

**DI-06 Carbon** (MV=missing value)

**Point**

1 MV 27.88 26.79 27.33 0.543

2 27.37 25.04 25.53 25.98 1.001

3 24.80 24.37 24.31 24.49 0.220

4 23.63 22.98 23.10 23.23 0.281

5 23.17 23.11 24.17 23.49 0.488

6 22.75 22.70 23.41 22.95 0.323

7 22.10 23.12 26.79 24.00 2.015

8 21.82 22.54 26.24 23.53 1.936

9 33.32 23.66 23.25 26.74 4.654

10 MV 25.22 26.36 25.79 0.572

**DI-06 Oxygen** (MV=missing value)

**Point**

1 MV 33.79 35.33 34.56 0.772

2 31.75 35.19 35.52 34.16 1.704

3 32.52 35.87 36.44 34.94 1.728

4 36.33 37.64 37.59 37.19 0.607

5 36.80 38.02 36.64 37.16 0.616

6 37.18 37.99 36.07 37.08 0.787

7 37.50 37.08 34.07 36.21 1.530

8 37.83 37.64 32.31 35.93 2.560

9 31.07 35.41 33.49 33.32 1.776

10 MV 34.33 30.83 32.58 1.750

**DI-06 Sodium** (MV=missing value)

**Point**

1 MV 0.43 0.45 0.44 0.006

2 0.37 0.47 0.44 0.43 0.042

3 0.42 0.56 0.56 0.51 0.066

4 0.51 0.61 0.57 0.56 0.039

5 0.54 0.57 0.53 0.55 0.015

6 0.56 0.52 0.53 0.54 0.016

7 0.47 0.54 0.45 0.49 0.040

8 0.35 0.45 0.43 0.41 0.043

9 0.27 0.40 0.41 0.36 0.064

10 MV 0.46 0.33 0.40 0.064

**DI-06 Magnesium** (MV=missing value)

**Point**

1 MV 0.68 0.75 0.71 0.037

2 0.55 0.62 0.61 0.59 0.030

3 0.52 0.62 0.59 0.57 0.041

4 0.55 0.54 0.35 0.48 0.094

5 0.53 0.35 0.38 0.42 0.080

6 0.44 0.32 0.76 0.51 0.185

7 0.35 0.37 0.42 0.38 0.030

8 0.32 0.47 0.53 0.44 0.086

9 0.24 0.36 0.86 0.49 0.270

10 MV 0.40 0.42 0.41 0.009

**Line A B C Mean SD**

**DI-06 Phosphorous** (MV=missing value)

**Point**

1 MV 12.66 12.43 12.55 0.115

2 13.22 13.34 13.27 13.27 0.051

3 14.09 13.42 13.44 13.65 0.309

4 13.54 13.32 13.19 13.35 0.141

5 13.57 13.05 13.07 13.23 0.243

6 13.43 13.13 13.92 13.49 0.327

7 13.47 13.27 12.94 13.23 0.221

8 13.34 13.40 13.80 13.51 0.207

9 11.64 13.55 14.76 13.31 1.283

10 MV 13.27 14.00 13.63 0.363

**DI-06 Chlorine** (MV=missing value)

**Point**

1 MV 0.02 0.03 0.03 0.004

2 0.04 0.03 0.02 0.03 0.006

3 0.03 0.03 0.04 0.04 0.006

4 0.03 0.05 0.04 0.04 0.007

5 0.05 0.05 0.05 0.05 0.004

6 0.05 0.07 0.05 0.06 0.007

7 0.09 0.08 0.10 0.09 0.007

8 0.09 0.07 0.10 0.09 0.012

9 0.24 0.08 0.07 0.13 0.075

10 MV 0.10 0.11 0.10 0.006

**DI-06 Potassium** (MV=missing value)

**Point**

1 MV 0.01 0.01 0.01 0.003

2 0.01 0.02 0.01 0.01 0.003

3 0.00 0.00 0.01 0.01 0.005

4 0.01 0.01 0.00 0.01 0.004

5 0.02 0.01 0.02 0.02 0.006

6 0.00 0.01 0.02 0.01 0.008

7 0.01 0.01 0.01 0.01 0.002

8 0.00 0.02 0.01 0.01 0.008

9 0.01 0.01 0.02 0.01 0.006

10 MV 0.00 0.02 0.01 0.010

**DI-06 Calcium** (MV=missing value)

**Point**

1 MV 24.53 24.21 24.37 0.164

2 26.70 25.29 24.60 25.53 0.874

3 27.62 25.13 24.60 25.78 1.317

4 25.40 24.84 25.15 25.13 0.229

5 25.31 24.83 25.13 25.09 0.198

6 25.59 25.26 25.23 25.36 0.163

7 26.01 25.53 25.23 25.59 0.323

8 26.25 25.42 26.58 26.08 0.487

9 23.22 26.52 27.14 25.62 1.721

10 MV 26.22 27.92 27.07 0.853

**Line A B Mean SD**

**DI-07 Carbon**

**Point**

1 24.55 25.62 25.08 0.533

2 25.11 26.84 25.97 0.867

3 30.47 27.79 29.13 1.343

4 28.99 26.94 27.97 1.023

5 27.81 28.54 28.18 0.364

6 29.38 28.73 29.05 0.328

7 29.54 27.96 28.75 0.791

8 29.11 26.99 28.05 1.063

9 29.49 24.19 26.84 2.652

10 30.54 24.85 27.70 2.845

**DI-07 Oxygen**

**Point**

1 33.40 32.86 33.13 0.268

2 33.73 33.36 33.55 0.184

3 32.79 33.67 33.23 0.441

4 31.49 30.48 30.98 0.503

5 31.79 30.73 31.26 0.531

6 30.49 31.44 30.96 0.472

7 29.28 31.80 30.54 1.256

8 31.66 33.87 32.76 1.104

9 31.09 33.87 32.48 1.388

10 31.31 34.77 33.04 1.732

**DI-07 Sodium**

**Point**

1 0.47 0.51 0.49 0.019

2 0.51 0.50 0.51 0.007

3 0.49 0.64 0.56 0.077

4 0.55 0.83 0.69 0.138

5 0.75 0.59 0.67 0.078

6 0.60 0.62 0.61 0.009

7 0.95 0.56 0.76 0.190

8 0.70 1.04 0.87 0.168

9 0.77 0.36 0.57 0.204

10 1.20 0.33 0.76 0.434

**DI-07 Magnesium**

**Point**

1 0.83 0.79 0.81 0.018

2 0.83 0.72 0.78 0.054

3 0.69 0.64 0.67 0.025

4 0.69 0.61 0.65 0.038

5 0.59 0.73 0.66 0.070

6 0.66 0.73 0.70 0.033

7 0.66 0.80 0.73 0.073

8 0.65 0.36 0.50 0.148

9 0.61 0.57 0.59 0.019

10 0.28 0.62 0.45 0.170

**Line A B Mean SD**

**DI-07 Phosphorous**

**Point**

1 13.47 13.36 13.41 0.057

2 13.56 13.03 13.29 0.268

3 12.14 12.62 12.38 0.241

4 12.94 13.76 13.35 0.410

5 13.17 13.21 13.19 0.021

6 13.07 12.97 13.02 0.048

7 13.34 13.15 13.24 0.096

8 12.82 12.67 12.74 0.075

9 12.87 13.78 13.32 0.455

10 12.47 13.59 13.03 0.560

**DI-07 Chlorine**

**Point**

1 0.02 0.02 0.02 0.005

2 0.03 0.03 0.03 0.002

3 0.04 0.03 0.03 0.002

4 0.02 0.04 0.03 0.011

5 0.03 0.03 0.03 0.001

6 0.05 0.02 0.03 0.016

7 0.03 0.03 0.03 0.001

8 0.06 0.03 0.04 0.015

9 0.03 0.03 0.03 0.001

10 0.07 0.01 0.04 0.031

**DI-07 Potassium**

**Point**

1 0.02 0.01 0.01 0.006

2 0.00 0.01 0.01 0.002

3 0.01 0.00 0.01 0.005

4 0.01 0.01 0.01 0.002

5 0.01 0.01 0.01 0.002

6 0.01 0.01 0.01 0.000

7 0.02 0.02 0.02 0.004

8 0.02 0.01 0.02 0.004

9 0.02 0.00 0.01 0.007

10 0.00 0.00 0.00 0.000

**DI-07 Calcium**

**Point**

1 27.24 26.84 27.04 0.199

2 26.23 25.51 25.87 0.356

3 23.37 24.60 23.99 0.617

4 25.30 27.32 26.31 1.007

5 25.86 26.16 26.01 0.152

6 25.74 25.50 25.62 0.122

7 26.18 25.68 25.93 0.247

8 24.98 25.05 25.01 0.033

9 25.12 27.20 26.16 1.041

10 24.13 25.82 24.98 0.847
